# REDUCING DYSFUNCTIONAL BELIEFS ABOUT SLEEP PROVIDES LONG-TERM BENEFIT IN OLDER ADULTS WITH INSOMNIA

**DOI:** 10.1093/geroni/igz038.1938

**Published:** 2019-11-08

**Authors:** Yeonsu Song, Constance Fung, Joseph Dzierzewski, Michael Mitchell, Karen Josephson, Lavinia Fiorentino, Jennifer Martin, Cathy Alessi

**Affiliations:** 1 School of Nursing, UCLA, Los Angeles, California, United States; 2 VA Greater Los Angeles Healthcare System, Los Angeles, California, United States; 3 Virginia Commonwealth University, Richmond, Virginia, United States; 4 VA Greater Los Angeles Healthcare System, North Hills, California, United States

## Abstract

Cognitive behavioral therapy for insomnia (CBTI) is recommended as first-line treatment in older adults. Changing dysfunctional beliefs and attitudes about sleep is an important component of CBTI, but the long-term impact of these changes are unknown, particularly in older adults. Methods involved secondary analyses of data from a large randomized controlled trial comparing CBTI (provided in 5 weekly sessions) to sleep education control, among older veterans with insomnia (N=159, mean age 72.2 years, 97% male, 79% non-Hispanic white). The purpose was to examine whether changes in a validated scale of Dysfunctional Beliefs and Attitudes about Sleep (DBAS) with CBTI treatment (baseline to post-treatment) was associated with later changes in self-reported sleep (post-treatment to 6 months follow-up). Sleep measures included Pittsburgh Sleep Quality Index (PSQI), Insomnia Severity Index (ISI), Epworth Sleepiness Scale (ESS) and 7-day sleep diary measures. Analyses compared the slope of change in DBAS (baseline to post-treatment) between CBTI and control with respect to the slope of change in sleep outcomes (post-treatment to 6-months). Compared to controls, the CBTI group had stronger associations between DBAS improvement (baseline to post-treatment) and subsequent PSQI improvement (post-treatment to 6-months) (difference in slopes=1.3, 95% CI=[.52,2.1], p=0.001). This pattern of significant results was also found for ISI (difference in slopes=1.8, 95% CI=[.58,3.0], p=0.004) and ESS (difference in slopes=1.0, 95% CI=[.25,1.7], p=0.009). Slopes were not different for sleep diary measures. These findings suggest that changing dysfunctional beliefs and attitudes may continue to confer sleep benefits well after completion of CBT-I in older adults.

